# Electrophysiological Assessment of the Effects of Obstructive Sleep Apnea on Cognition

**DOI:** 10.1371/journal.pone.0090647

**Published:** 2014-02-28

**Authors:** Ethem Gelir, Cenk Başaran, Sibel Bayrak, Süha Yağcıoğlu, Murat Timur Budak, Hikmet Fırat, Pekcan Ungan

**Affiliations:** 1 Department of Physiology, Hacettepe University Medical School, Ankara, Turkey; 2 Department of Biophysics, Hacettepe University Medical School, Ankara, Turkey; 3 Sleep Laboratory, Department of Pulmonary Medicine, SGK Ankara Education Hospital, Ankara, Turkey; 4 Department of Biophysics, Koç University Medical School, İstanbul, Turkey; Hospital General Dr. Manuel Gea González, Mexico

## Abstract

We used electrophysiological measures to investigate the effects of obstructive sleep apnea on attention, learning, and memory. Thirty subjects (OSA group, *n* = 15, control group *n* = 15) participated in *n*-back tests, accompanied by P300 recordings, to investigate working memory and attention. The mirror-drawing test was used to study procedural memory, and the trail-making test (TMT) was used to evaluate divided attention and executive function. No significant group difference in reaction time was found in the 0-back and 1-back tests. In the 2-back test, reaction times of patients were longer than those of the control group. No P300 wave was obtained in the OSA group in any (0-, 1-, or 2-back) *n*-back test. In contrast, in the control group, significant P300 waves were recorded except for the 2-back test. The mirror-drawing scores were unaffected by sleep apnea. There was no difference between groups in the TMT-A test on any of the trials. Although no group difference was found in the first or second trials of the TMT-B test, OSA patients were less successful in learning on the third trial. According to our study results, OSA affects attention and executive function adversely however, we could not detect a significant effect on working or procedural memory.

## Introduction

Obstructive sleep apnea (OSA) is a condition characterized by episodes of sleep apnea (cessation of breathing 10 s or longer) or hypopnea (significant reduction in breathing), oxygen desaturation, and frequent arousal [1]. It is one of the most common sleep disorders, found in at least 2–4% of subjects 30–60 years old [2]. Untreated OSA is associated with cardiovascular, metabolic, and cerebrovascular morbidity [1], [3]. OSA patients may have respiratory disturbances, such as hypoxemia and hypercapnia due to airway occlusion. Additionally, a series of neuropsychological deficits has been reported in OSA patients, including impairment in working memory (WM) [4] and long-term memory [5], alterations in a wide spectrum of attentional processes [6], and dysfunction in cognitive executive function [7]. Hypoxemia associated with OSA may be a factor contributing to this neuropsychological impairment.

However, the results of studies on executive function in OSA syndrome are varied. Some studies have reported diminished or impaired cognition, executive function, and attention [8]–[12], whereas others have not [13]–[16]. One method that has been extensively used to assess cognitive and attentional deficits is measurements of event-related potentials (ERP). The most common tasks used in ERP studies is the “oddball paradigm,” in which subjects are requested to respond to target stimuli randomly scattered among non-target ones. [17]. P300 characteristics (i.e., amplitude and latency) have been evaluated using visual or auditory oddball paradigms in a few ERP studies of OSA patients. However, the interpretation of P300 results is controversial [18]–[22].

Distinct aspects of cognition are assessed by different tests (e.g., *n*-back task, trail-making test, mirror-drawing test) in OSA patients. Memory is usually assessed by the *n*-back task, which requires maintenance of the previous *n* stimuli in WM until the present response can be executed [12]. The trail-making test (TMT) is widely used to test executive function, such as complex attention, planning, cognitive set shifting, and response inhibition [23]. The mirror-drawing (MD) test, another commonly used method, analyzes implicit learning abilities and requires fast and repetitive processing of visuospatial stimuli but no acquisition of sequence [24].

Our review of the literature found no study that assessed both electrophysiological and behavioral aspects of cognitive functioning in OSA patients. The aim of this study was to investigate the electrophysiological indications of the effects of obstructive sleep apnea on attention, learning, and memory. We also assessed the relationship of these cognitive characteristics with the apnea-hypopnea index (AHI) and overnight mean O_2_ saturation (SpO_2_mean). We believe this is the first reported study to extensively assess cognitive and attentional deficits in OSA patients.

## Methods

### Ethics Statement

Hacettepe University Institutional Ethical Committee approved the study, and all procedures were carried out according to the Declaration of Helsinki. All subjects provided written informed consent prior to participating in the study.

### Experimental Design

All subjects spent one night in the sleep laboratory with polysomnography. The polysomnography setup began at 21∶00 and lights-off time occurred between 22∶00 and midnight. The next morning, participants were awakened at 6∶00 and polysomnography recording equipment was removed. Upon completion of polysomnography recordings, participants meeting all criteria were scheduled to return at a later date (within 2 weeks) for behavioral and electrophysiological tests. At the behavioral test day, we started to record ERPs simultaneously during n-back tasks at 13∶30. It took approximately 1 hour to complete these two tests. After 15 minute break, we continued with the mirror-drawing test and gave another 15 minute break. We completed the behavioral tests with the trail-making A and B tests.

### Participants

We prospectively selected full-night polysomnographic studies from the patients’ record registry of Ankara Diskapi Sleep Clinic. Fifteen newly diagnosed OSA patients and 15 non-apneic controls were enrolled in this study. Cigarette smoking status, history of chronic diseases, drugs, and habits were obtained using a standardized questionnaire before the sleep study. Sleepiness was assessed by the Epworth Sleepiness Scale. In total, 30 volunteer male subjects participated. Participants with an apnea–hypopnea index higher than 18 were assigned to the “OSA” group (*n* = 15), and participants with an apnea–hypopnea index lower than 5 were assigned to the “control” group (*n* = 15). Participants in both groups were right-handed with no co-morbidities, and no past treatment for OSA. The groups were matched for age. Participants were also selected to minimize any education effect by requiring that they had graduated from elementary school and had no education beyond high school. Exclusion criteria were the presence of any significant deviation from average in polysomnographic sleep parameters, clinically significant medical disorders (i.e., neurological or psychiatric diseases including depression, primary perception disorder), the use of drugs known to affect sleep or daytime sleepiness, and substance or alcohol abuse. Fasting blood samples were taken for complete blood count and liver, renal and, thyroid function tests as routine tests for all subjects. However, we did not test participants for alcohol and illicit drugs. Subjects were instructed to refrain from ingesting any caffeinated beverages in the 12 h prior to measurements.

### Polysomnography

All participants underwent standardized sleep center clinical procedures. Standard surface electrodes were used to record electroencephalographic (four EEG derivations: C3, C4, O1, and O2), electrooculographic, electromyographic (submental and anterior tibialis muscles), and electrocardiographic data. Nasal–oral thermocouples were used to monitor airflow, and thoracic and abdominal movements were used as indications of respiratory effort. Blood oxygen saturation was monitored with pulse oximetry (with the sensor placed on the earlobe). Sleep latency, sleep efficiency, percentage of sleep time in each sleep stage, and other polysomnographic parameters were calculated. Currently published standard techniques for humans were used for recording and scoring, including procedures for sleep stages [25], [26] and leg movement [27].

### Behavioral and Electrophysiological Protocols

All subjects performed three sessions of visuo-spatial *n*-back tests (0-back, 1-back, and 2-back). Event-related potentials were recorded simultaneously during *n*-back tasks. Subsequently, subjects were asked to complete mirror-drawing and TMT-A and TMT-B tests. Prior studies demonstrated that both circadian phase and time awake are important factors influencing mental performance. In order to control for potential circadian rhythm effects, experimental protocols were always conducted between 13∶30 and 17∶30.

### *N*-back Test

We used a visually presented verbal *n*-back sequential letter task to probe working memory. In this task, subjects saw a string of upper or lower case letters (A, B, C, D, E, H), presented at a rate of one every 2300 ms, with each letter displayed for 200 ms. Three conditions were presented: 0-back, 1-back, and 2-back. On every trial, the subjects had to make a decision as to whether the current letter matched according to the rule, which changed between blocks. Strings of letters occurred in a pseudo-random order such that the same condition did not occur twice in a row. After a concise introduction of the *n*-back task, all participants were trained prior to actual tests until they reach 70% accuracy on the one-back task. All participants achieved this in their first attempt. Subjects were asked to decide whether the current letter matched a single target letter that was specified before the block began. The 0-back condition required that participants press a button each time the same letter seen (e.g., “H”) was presented in the series of letters [28]. Under the 1-back condition, they were asked to decide whether the current letter matched the previous letter. Under the 2-back condition, the task was to decide whether the currently presented letter matched the letter that had been presented two back in the sequence. Subjects responded to all items with a button press. Experimental tasks were presented by PC interface and were programmed using the STIM 2.0 software package (Compumedics Neuroscan, Inc., El Paso, TX). The PC also recorded subjects’ responses and reaction times (RTs) in s. The *n*-back tests lasted approximately 40 min, including short breaks. The 1-back condition consisted of 160 trials with 48 matches (30% hit rate) and never contained more than one consecutive hit in a row. The 2-back condition consisted of 250 trials with 60 matches (24% hit rate) and never contained more than two consecutive hits in a row.

### P300 Recording

P300 experiments were conducted in an acoustically and electrically shielded chamber. Event-related potentials (ERPs) were recorded using a Neuroscan NuAmps (Compumedics) digital amplifier. ERPs were recorded from Fz, Pz, Cz, and O1 scalp electrodes using the Electro-Cap System (Eaton, OH) and referenced to linked earlobe electrodes. Impedances were kept below 10 kΩ. The sampling rate was 1000 Hz. The EEG was band-pass filtered between 0.5 and 70 Hz. An online band-pass filter (1–30 Hz) was also used. Data were epoched from 0 ms pre-stimulus to 1000 ms post-stimulus. Eye-blink artifacts were monitored using two electrodes just above and below the right eye. Epochs in which the amplitude exceeded ±100 µV in any channel were rejected automatically. The amplitude used in our study was calculated relative to absolute zero.

### Mirror-drawing Test

The subjects participated in three successive test conditions within a single experimental session. The mirror was located at the left (front) of the sheet of paper and was placed perpendicular to the graphic workplane. During the tests, a mask prevented the subjects from having a direct view of the model or of their drawing movements. Subjects were asked to use a pencil to trace a shape while looking at it in a mirror. Five templates used in the study were straight-line shapes with horizontal and oblique lines forming 2, 4, 6, 8, and 10 corners. All templates had a total line length of 22 cm and a width between guide lines of 6.6 mm.

Subjects immediately traced the template, under instruction to move as fast and accurately as possible, attempting to stay within the line of templates. At the end of the line, they lifted the pen away from the paper, providing a clear indication of the end of their tracing action. Templates were presented in pseudorandom order, balanced across control subjects, with three repetitions of the five basic shapes (15 total trials). The time (s) to complete the task was recorded as the initial score for the mirror-drawing tests. Some subjects crossed the borders even though they completed the test. To equalize the scores of subjects who completed the test quickly but with errors and those of subjects who did the test more slowly with no errors, initial score were recalculated by dividing the completion time by the percentage of completed drawing within the total area.

### Trail-making Test

The TM test had two parts. In Part A, the participant was asked to connect disordered circles enclosing the numbers 1 to 25 on the test form with direct lines to obtain a correct sequential alignment. Similarly, in Part B, the participant was asked to connect disordered circles enclosing both numbers from 1 to 13 and letters from A to L on the test form with direct lines to obtain a correct alignment such that the corresponding number followed each letter (i.e., 1-A, 2-B, 3-C, *etc*.). The testing equipment included four A4-size sheets of paper and a pencil. The first sheet was the practice page for Part A, and the second was the test page for Part A; similarly, the third sheet was the practice page for Part B, and the last was the test page for Part B. All tests were repeated three times, and the averages of measurements were used for statistical analyses. Score A, the time spent to complete Part A, and Score B, the time spent to complete Part B, both in s, were determined by the tester with the help of a digital hand chronometer. In addition to these two scores, we also used two derivative scores previously proposed [29] to evaluate the subjects. The derivative parameters were Score B – A (difference between time spent completing Parts B and A, in s) and Score A+B (sum of time taken to complete Parts A and B, in s).

### Statistical Analysis

Statistical analyses were performed using GraphPad InStat 3.1 and Prism 5.03 (GraphPad Software, San Diego, CA, USA). The Kolmogorov–Smirnov test was used to analyze the normal distribution of the variables (*P*>0.05). The age and BMI of subjects and numerical test results of polysomnographic data, amplitude of P300 wave, *n*-back performance, and mirror-drawing and trail-making test results of the two groups were compared using the unpaired Student’s *t*-test. Intra-group comparisons of *n*-back results and TMT-A and -B results were analyzed by repeated-measures analysis of variance (ANOVA) followed by a *post hoc* Tukey test. *P*-values less than 0.05 were considered to indicate statistical significance. Correlations between numerical variables were determined using Spearman’s correlation coefficient.

## Results

### Clinical and Behavioral Measurements

Table 1 summarizes the polysomnographic data. All the patients and controls were men. The patients’ mean age was 41.5±2.5 (range, 26–53) years, and that of the controls was 35.8±2.5 (range, 27–50) years. The groups were not statistically significantly different in terms of age, body mass index (BMI), SpO_2_mean, percentage of rapid eye movement (REM) sleep, or total sleep time (TST). AHI and pulse oxygen saturation nadir in sleep (SpO_2_nadir) percentage in the OSA group were significantly different from those for the control group (*P*<0.05; Table 1).

**Table 1:** Subject characteristics.

Demographic characteristics and sleep parameters	Control (*n* = 15)	OSA (*n* = 15)
**Age**	35.9±2.5	41.5±2.5
**BMI**	28.1±1.1	30.7±1.1
**AHI**	3.0±0.5	36.1±6.9*
**SpO_2_mean (%)**	91.5±1.5	91.1±0.9
**SpO_2_nadir (%)**	83.5±3.4	77.2±3.0*
**REM sleep (%)**	15.8±1.2	12.3±1.1
**TST (min)**	388.1±9.8	374.7±6.9

Values are presented as means ± SEM.

AHI = apnea–hypopnea index, REM = rapid eye movement, TST = total sleep time.

*Significantly different compared with control group, *P*<0.05.

### The *n*-back Test

The *n*-back test results are given in Table 2. There was a decline in performance accuracy and an increase in the RT as we increased the working memory load in both control and OSA groups (*p*<0.05). No significant difference was found between the accuracy values of the OSA and control groups at any level of the task load. Only RT of the 2-back test was significantly different between the control and OSA groups (*p*<0.05). There was no correlation between *n*-back test results and age or AHI (*p*>0.05). SpO_2_mean and SpO_2_nadir values were positively correlated with the number of incorrect responses during the *n*-back test at the 0-back level (*r* = 0.535, *p*<0.05 and *r* = 0.579, *p*<0.05, respectively).

**Table 2:** *N*-back task behavioral performance.

	Control (*n* = 15)	OSA (*n* = 15)
***N*** **-back Performance (s)**		
0-back, percentage correct	92.6±2.6†	95.4±0.8¥
1-back, percentage correct	89.2±3.5†	91.4±1.7¥
2-back, percentage correct	62.5±7.0	63.1±6.8
**Reaction Time (s)**		
0-back RT	382.2±17.8	409.4±23.7
1-back RT	449.7±31.5‡	511.2±27.0§
2-back RT	473.2±29.3‡	591.4±43.8* §

*N*-back performance and reaction times are presented as means ± SEM.

*p<0.05, vs. Control.

† *p*<0.001, vs. 2-back, percentage correct (Control).

‡ *p*<0.05, vs. 0-back RT (Control).

¥ *p*<0.001, vs. 2-back, percentage correct (OSA).

§ *p*<0.05, vs. 0-back RT (OSA).

### P300

**Figure 1:**
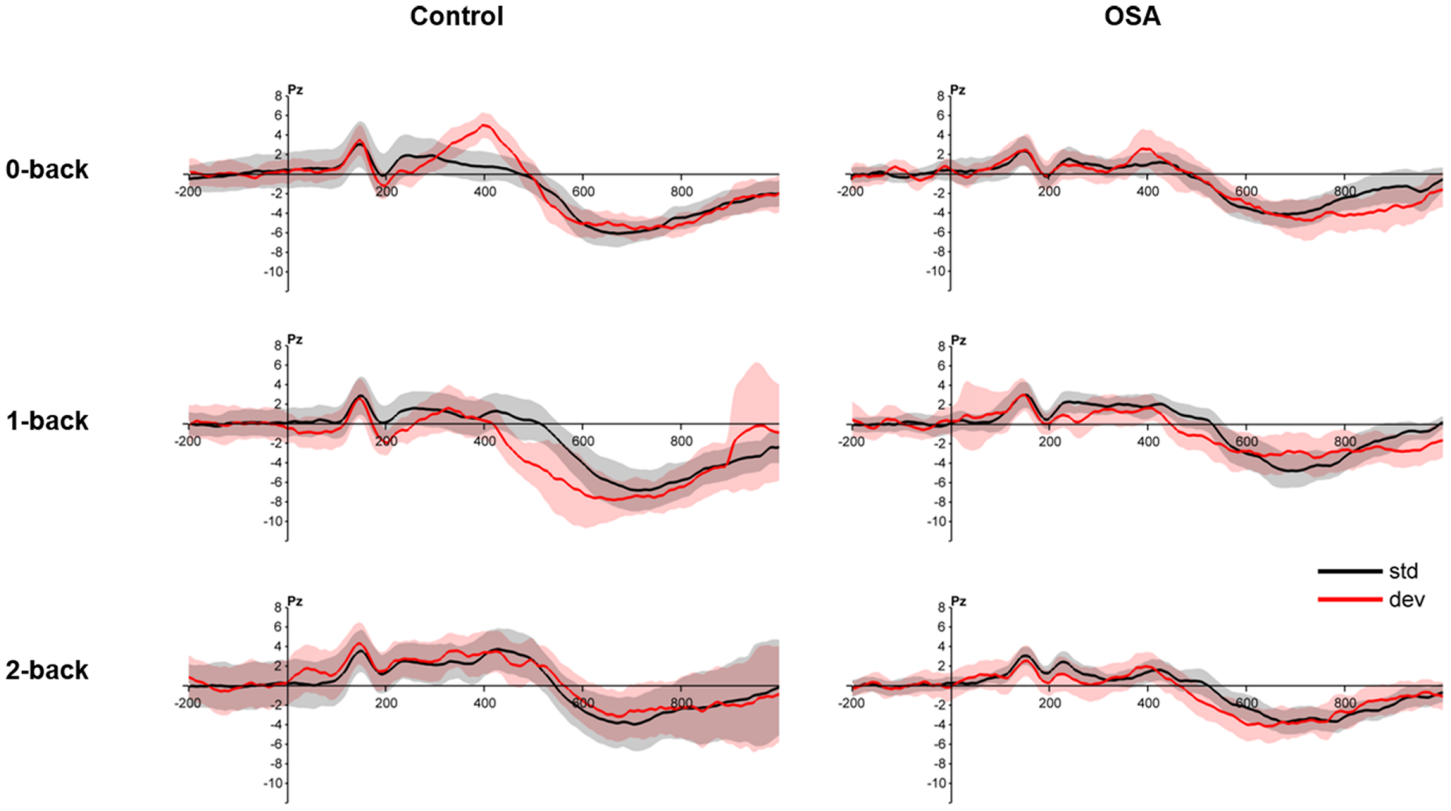
Statistically significant P300 waves were recorded only under the 0-back condition. Grand average for subjects in control and OSA groups. Top row: control, bottom row: OSA. Results are shown for the Pz electrode. Black: responses to standard (non-target) stimuli, red: responses to deviant (target) stimuli. Shaded regions indicate the confidence interval of the responses (mean ±2 SEM).

Results of the *n*-back P300 recordings are shown in Figure 1. P300 waves could only be recorded in the 0-back condition. The latency of the P300 wave was 400 ms. In the control group, the mean difference between the peak amplitudes of the deviant and standard responses was 4.3 µV. The standard error of this mean was 0.6 µV, and the difference was statistically significant (*t* = 7.5, *p*<0.001, *df* = 16). For the patient group, the mean and the standard error of the mean for this difference were 1.6 µV and 1.0 µV, respectively, which was close to being significant (*t* = 1.6, *p* = 0.14, *df* = 16). The mean peak amplitude of the P300 wave for the control group was 2.7 µV greater than that for the patient group (S.E. of the difference = 1.2, *t* = 2.2, *p*<0.05, df = 24.8).

### Mirror-drawing and Trail-making Tests

No statistically significant difference was found between the groups in the mirror-drawing test (data not shown). TMT results are provided in Table 3. When the groups were compared, only the TMT-B third trial showed a significant difference (*p*<0.05); no statistically significant difference was found for any TMT-A trial or the first or second trials of TMT-B. TMT performance improved generally in both groups over the trials (*p*<0.05).

**Table 3:** Trail-making test results.

	Control (*n* = 15)	OSA (*n* = 15)
**TMT-A1 time (s)**	23.4±1.5†	30.2±3.2
**TMT-B1 time (s)**	74.7±9.7	72.4±7.5
**B1-A1 (s)**	51.3±8.6	42.2±6.7
**B1/A1 (s)**	3.2±0.3	2.5±0.2
**TMT-A2 time (s)**	22.8±2.4†	24.3±1.9¥
**TMT-B2 time (s)**	60.9±8.2‡	59.4±4.6§
**B2-A2 (s)**	38.0±6.7	35.1±3.8
**B2/A2 (s)**	2.7±0.3	2.5±0.2
**TMT-A3 time (s)**	18.2±1.3	21.180±1.8¥
**TMT-B3 time (s)**	42.7±2.9‡	64.9±7.1* §
**B3-A3 (s)**	33.6±8.9	43.8±5.6
**B3/A3 (s)**	2.890±0.4	3.078±0.1

TMT-A and TMT-B values are given as means ± SEM.

*p<0.05, vs. Control.

† *p*<0.05, vs. TMT-A3 (Control).

‡ *p*<0.05, vs. TMT-B1 (Control).

¥ *p*<0.01, vs. TMT-A1 (OSA).

§ *p*<0.001, vs. TMT-B1 OSA.

## Discussion

The aim of the current study was to examine the effects of OSA on cognitive ability. We also sought to understand any association between hypoxemia and cognitive performance in patients with OSA. There was no difference between the groups in *n*-back tests at any level of the task load. However, similar to the results reported by Thomas et al., we also found that the 2 -back response time in the OSA group was longer than that in the control group. However, the 2-back results were not correlated with AHI or SpO_2_ in our study or in others [30], [31]. Moreover, we found no correlation between age and the 2-back test results. Thomas et al. had similar results; they also reported that the 2-back test results were not correlated with age or BMI [30].

The P300 component is considered an index of general cognitive functioning or attention resources [17], [32], [33]. Previous studies using event-related potentials (ERP) have consistently shown changes in P300 suggesting attention deficits in OSA [34]. Most previous studies used 0-back tests in ERP studies. In 0-back tests, we measured the amplitude of the P300 wave as 1.6 µV in the OSA group and 4.3 µV in the control group. Our results suggest that the P300 changes observed in OSA are associated with decreased vigilance. In 1-back and 2-back tests, we could not detect a meaningful P300 wave in either group because of the increased memory load.

The mirror-drawing test results were unaffected by OSA, similar to the findings of Rouleau et al. [35]. The mirror-drawing test has been shown to be significantly affected by age, and this is interpreted as consumption of cognitive sources [36]. Our OSA parameters were unrelated to mirror-drawing test performance, perhaps because all of our subjects were relatively young. Possibly in older subjects, apnea-induced hypoxia, along with other age-related neurological problems, may cause deterioration in procedural function and working memory.

Our trail-making test results showed no difference between the groups in TMT-A. However, the OSA group was slower than the control group in trail making test B. No reported study has shown a deficiency in TMT-A in OSA patients [15]. The results for TMT*-*B vary. Some studies have reported no deficiency [37], whereas others have found the opposite [14], [35]. Although trail-making tests have typically been conducted once in previous studies, we repeated the tests three times and investigated the effect of OSA on learning ability. According to our results, there was no difference between the two groups at the beginning, but in subsequent tests, we found that control group Score B (time spent to complete Part B in s) was significantly shorter relative to that of the OSA group**.** This finding suggests that the learning process was negatively affected in the OSA group. Some declarative memory components of the trail making test differ from the mirror-drawing test. Because of this, OSA patients may have difficulty in the trail-making test, although they have no problem in the mirror-drawing test.

In summary, according to our study results, OSA does not appear to affect cognitive function as measured by the *n*-back test (working memory) and mirror-drawing test (procedural memory). However, in the 0-back test, the amplitude of the P300 (attention) was smaller in the OSA group than in the control group, and TMT-B (executive function) results in OSA patients were significantly slower than those in the control group. These results suggest that OSA affects attention and executive function adversely. In this study, we did not consider the duration or severity of OSA, which might affect the results. We do not think that the connection between OSA and the deterioration in cognitive function is a simple cause-and-effect relationship. Other factors in addition to OSA and age may be involved; the best candidate is genetics. For example, the APOE4 gene, a risk factor for Alzheimer disease, is also a risk factor for OSA. We did not investigate this gene in our subjects. In the light of our results and those of other studies, we think that research should be supported by genetic studies because age and OSA severity alone would not seem to sufficiently explain this connection.
